# Higher Serum Neuropeptide Y Levels Are Associated with Metabolically Unhealthy Obesity in Obese Chinese Adults: A Cross-Sectional Study

**DOI:** 10.1155/2020/7903140

**Published:** 2020-08-04

**Authors:** Hao-Neng Tang, Fen Xiao, Ya-Ru Chen, Si-Qi Zhuang, Yue Guo, Hui-Xuan Wu, Hou-De Zhou

**Affiliations:** ^1^Department of Metabolism and Endocrinology, Hunan Provincial Key Laboratory for Metabolic Bone Diseases, National Clinical Research Center for Metabolic Diseases, The Second Xiangya Hospital of Central South University, Changsha, Hunan 410011, China; ^2^Department of Laboratory Medicine, The Second XiangYa Hospital of Central South University, Changsha, Hunan 410011, China

## Abstract

**Objective:**

Neuropeptide Y (NPY), an orexigenic peptide known to cause hyperphagia, has been involved in the occurrence and development of obesity. However, differences in the distribution of serum NPY levels in obese phenotypes (including metabolically unhealthy obesity (MUO) phenotype and metabolically healthy obesity (MHO) phenotype) and the association of NPY with MUO phenotype have not been unequivocally established. We therefore determined associations of serum NPY levels with MUO phenotype in obese Chinese adults.

**Methods:**

A cross-sectional study was conducted from 400 obese adults in Hunan province, who underwent a health examination in the Second Xiangya Hospital, and 164 participants were finally enrolled in the study and divided into MHO and MUO groups. Serum NPY levels were examined; univariate and multivariate analyses as well as smooth curve fitting analyses were conducted to measure the association of NPY serum levels with the MUO phenotype.

**Results:**

Serum NPY levels were significantly elevated in the MUO group compared with the MHO group ((667.69 ± 292.90) pg/mL vs. (478.89 ± 145.53) pg/mL, *p* < 0.001). A threshold and nonlinear association between serum NPY levels and MUO was found (*p* = 0.001). When serum NPY levels exceeded the turning point (471.5 pg/mL), each 10 pg/mL increment in the NPY serum level was significantly associated with an 18% increased odds ratio of MUO phenotype (OR: 1.18, 95% CI: 1.07–1.29, *p* = 0.0007) after adjusted for confounders.

**Conclusions:**

Higher NPY serum levels were positively correlated with MUO phenotype in obese Chinese adults.

## 1. Introduction

Obesity is a multifactorial and chronic disease mainly caused by excess energy intake, which has caused a major global public health problem [1–3]. Life expectancy is expected to decrease as the obesity prevalence rates soar [4, 5]. More importantly, obesity typically develops because of small but long-term changes in energy balance, which causes a series of energy metabolism disorders [2]. Hence, obesity may increase the risk of premature death because of the significantly increased risk for hypertension, type 2 diabetes mellitus (T2DM), cardio-cerebrovascular diseases, and some cancers [2, 5, 6].

However, metabolic disorders are not necessarily triggered when body mass index (BMI) reaches a level indicating overweight or obesity [7, 8]. Hence, it makes sense to distinguish obese individuals with metabolic disorders from those who are metabolically healthy [5]. Obesity has two major phenotypes depending on whether it is accompanied by metabolic abnormalities. Metabolically healthy obesity (MHO) refers to an obesity phenotype with little or no metabolic abnormalities [2], while the metabolically unhealthy obesity (MUO) phenotype has at least two or more concomitant metabolic disorders [9]. There is increasing evidence that patients with MUO are more prone to have cardiovascular events than whose with MHO, but the MHO phenotype is still more likely to develop MUO phenotype than healthy people with normal weight [1, 7–9]. Thus, it is vital to identify further metabolic abnormalities in people with obesity and to explore additional risk factors.

Neuropeptide Y (NPY), one of the most powerful orexigenic peptides, is produced in large amounts in the arcuate and dorsal medial nucleus of the hypothalamus, as well as in peripheral adipose tissue [10–12]. Excess release of NPY can lead to overeating causing excessive energy intake, subsequently precipitating a series of damaging effects resulting in diabetes, heart disease, and other illnesses [11, 13]. Our previous study [14] and other studies have found that NPY regulates fat metabolism by promoting adipocyte differentiation [15, 16], inhibiting lipolysis [17], and suppressing the beigeing in white adipose tissue [18]. Overexpression of NPY in mice has been reported to cause the obesity phenotype and insulin resistance, leading to a series of metabolic disorders [19], while the use of NPY antagonists has been found to reduce obesity and metabolic imbalances in fat tissue associated with aging [17]. Therefore, NPY has an important relationship with the occurrence and development of obesity and its related complications.

Many obesity models were characterized by an increase in central nervous system NPY tone, which lead to overeating and obesity [20]. However, NPY is also involved in the development of metabolic disorders without causing overeating in mouse models [21]. Although several studies have reported the contributions of NPY to obesity [22, 23] and T2DM [24], none of them have assessed the specific effects of NPY serum levels on obesity in distinct metabolic conditions. Furthermore, research on differences in NPY levels between various obesity phenotypes has not been published.

Given that neuropeptide Y is a key orexigenic peptide affecting on obesity, we hypothesized that high serum NPY level is an essential risk factor for the MUO phenotype, and the main purpose of the present study was to investigate differences in NPY serum levels between individuals with various obesity phenotypes and to analyze the specific association of NPY with MUO.

## 2. Materials and Methods

### 2.1. Study Subjects

A cross-sectional study was performed with 400 obese participants (aged 19 to 78 years) who were screened based on their body mass index (BMI, BMI ≥25.0 kg/m^2^) [25], which was measured during a physical examination between November 2018 and February 2019 in the Second Xiangya Hospital of Central South University in China. All participants have signed informed consent form. The study was approved by the ethics committee of the Second Xiangya Hospital of Central South University (Clinicaltrials.gov ID: ChiCTR-EOC-16010194).

The major exclusion criteria in this study included (1) cancer, hematologic disease, organic heart disease, autoimmune disease, hyperthyroidism, Cushing syndrome, hyperparathyroidism, infection, history of myocardial infarction and stroke, history of tuberculosis, chronic alcohol abuse, pregnancy, and lactation; (2) diseases that may cause abnormal levels of NPY, such as psychiatric disorders and epilepsy; (3) subjects with unqualified specimens (e.g., hemolysis); and (4) missing data on anthropometric data and metabolic components. The sample size was estimated based on confidence level (1-*α*), power of test (*β*), tolerance (*δ*), and sample standard deviation (s) [26].To estimate the sample size, a preliminary pilot study was conducted for measuring the serum NPY levels in 40 obese participants, and the sample SD was 179.6 pg/mL. Since the confidence level was 0.95, and the tolerance was 5%, a sample size of 152 individuals was estimated based on these parameters. In this study, 164 participants were eventually included based on all exclusion criteria (*n* = 236). These obese subjects were subsequently classified as two subgroups based on the presence/absence of metabolic abnormalities. Nonobese ATP III criteria [27] of components of metabolic syndrome was used for identification of metabolic abnormalities (TG ≥ 1.7 mmol/L, HDL − C < 1.04 mmol/L in men, HDL < 1.29 mmol/L in women, FBS > 5.6 mmol/L, and SBP/DBP > 130/85 mmHg) [28]. The presence of ≥2 ATP III components of metabolic syndrome in participants was defined as MUO (*n* = 93), and the presence of ≤1 component was defined as MHO (*n* = 71) [2, 9].

### 2.2. Data Collection

The anthropometric data that were collected mainly included BMI, waist circumference (WC), and waist hip rate (WHR). Systolic and diastolic blood pressure (SBP, DBP) were measured with the participant in a seated position and resting state.

All blood samples were obtained at 8 am after fasting for 10-12 hours and centrifuged at 3,000g for 5 min to separate the serum. Serum levels of triglycerides (TG), total cholesterol (TC), high-density lipoprotein cholesterol (HDL-C), low-density lipoprotein cholesterol (LDL-C), nonesterified fatty acid (NEFA), fasting blood sugar (FBS), and whole blood levels of HbA1c were tested in the Department of Laboratory Medicine of the Second Xiangya Hospital, using routine diagnostic tests [29]. Serum NPY levels (CEA879Hu 96T, CLOUD-CLONE CORP, USA) were measured with specific enzyme-linked immunosorbent assays (ELISA) with an intra-assay error <10% and an interassay error <12%.

### 2.3. Statistical Analysis

We described characteristics of the subjects using means and standard deviations (SD) for normally distributed variables, median and interquartile range (IQR) for nonnormally distributed variables, and percentages for categorical variables. Independent samples *t*-test, Mann-Whitney *U* test, and *χ*^2^ test were used for comparison of normally, nonnormally distributed continuous variables, and categorical variables, respectively. Spearman's correlation coefficient was performed for evaluating the relationship of NPY with clinic-metabolic parameters.

Univariate analyses and multiple logistic regression were used to determine the association between NPY serum levels and MUO phenotype. We began with an unadjusted model and then added some confounders defined as covariates based on their clinical implication and significance in univariate analysis (*p* < 0.15). NPY serum levels were further treated as both continuous variables in increments of 10 pg/mL and categorical variables in quartiles in all models. *p* values for trend were also obtained by coding NPY level categories (quartiles) as ordinal variables in the regression models. Stratified analyses and interaction analyses were further performed by gender (male, female), age (<45 years, ≥45 years), and BMI (25–28 kg/m^2^, ≥28 kg/m^2^) to confirm the relationship between NPY serum levels and the MUO phenotype in the obese participants.

To explore the potential nonlinear association between NPY and MUO phenotype, smooth curve fitting analysis and a piecewise linear regression model were performed to further examine the threshold effect of NPY on MUO. All analyses were performed using Empower (R) [30]. All tests were based on a two-tailed *p* value of less than 0.05 as evidence of statistical significance.

## 3. Results

### 3.1. Clinical and Laboratory Characteristics of the Participants

This analysis included 164 participants (44.53 ± 14.04 years, 73% men) of whom 43.3% and 56.7% had MHO and MUO, respectively. The baseline data of the participants were presented in Table 1. A significant difference in age was found between MHO and MUO groups, with the mean age (1 SD) of the MHO group was 39.94 (13.09) years and 48.03 (13.78) years for the MUO group. There was no significant difference in gender composition between the MHO group and MUO group, and males made up the larger percentage of both groups (70.4% of the MHO group and 75.3% of the MUO group). Compared with the MHO group, participants in the MUO group had higher WC, SBP, DBP, TG, LDL-C, TC, FBS, and HbA1c levels and lower HDL-C levels (all *p* < 0.05).

As for NPY serum levels, the trend in the distribution of the expression of NPY was similar to that of the major metabolic indicators (i.e., TG, TC, FBS, SBP, DBP, and LDL-C) between these two groups. Serum NPY level was significantly increased in MUO group compared with MHO group (667.69 (292.90) pg/mL vs. 478.89 (145.53) pg/mL, *p* < 0.001).

### 3.2. Univariate and Multivariate Regression Analyses of MUO's Association with Serum NPY Levels

We first conducted univariate analyses to analyze the relationship between MUO and each variable. As shown in Table 2, positive associations were found between MUO and BMI, WC, WHR, SBP, DBP, TG, TC, LDL-C, FBS, HbA1c, and LDL-C (all *p* < 0.05), whereas a negative association was observed between MUO and HDL-C (*p* < 0.001).

Multivariate regression analyses were then conducted to analyze the specific association of serum NPY levels with MUO (Table 3). The association of NPY with MUO was measured by the odds ratio (OR). Our results showed that per 10 pg/mL increment in NPY was associated with higher risk of MUO (OR: 1.06, 95% CI: 1.03–1.08, *p* < 0.0001) in the linear modeling before adjusting for confounders (model 1). After adjusted for sex, age, BMI, WC, WHR, TC, LDL-C, and HbA1c (model 4), higher level of NPY was still independently associated with MUO (OR: 1.07, 95% CI: 1.03–1.12, *p* = 0.001).

We also observed evidence of a dose-response trend across NPY quartiles (*p* for trend < 0.0001) in all the models. The ORs for MUO were significantly higher in the upper quartile of serum NPY levels than in the lower quartile. The third and fourth quartiles of NPY levels were associated with a 1.75 (OR: 2.75, 95% CI: 1.12–6.78, *p* = 0.0278) and 18.92 (OR: 19.92, 95% CI: 5.86–67.71, *p* < 0.0001) times higher risk of MUO compared with the first quartile in the crude model (model 1), respectively. In the analyses adjusted for sex, age, BMI, WC, and WHR (model 3), the ORs were gradually increased for MUO; the third and fourth quartiles of NPY levels were associated with a 4.41 (OR: 5.41, 95% CI: 1.15–25.39, *p* = 0.0324) and 37.99 (OR: 38.99, 95% CI: 6.18–246.01, *p* < 0.0001) times higher risk of MUO compared with the first quartile. After further adjusting for TC, LDL-C, and HbA1c (model 4), we observed that the fourth quartile of NPY was significantly positively associated with the MUO (OR: 29.85, 95% CI: 4.38–203.62, *p* = 0.0005).

Further stratified analyses and interaction analyses were conducted by sex, age, and BMI (Table 4). A gender difference and age difference were observed in the association between NPY and MUO. The level of NPY being positively associated with MUO in the male sample (*p* = 0.0039) and in participants with age ≤45 years (*p* = 0.0041), but the association was insignificant in the female sample (*p* = 0.1906) and in participants with age >45 years (*p* = 0.1603). At different BMIs, NPY levels showed a positive association with MUO (25–28 kg/m^2^, *p* = 0.0166; ≥28 kg/m^2^, *p* = 0.0078). An interaction analysis revealed that the variables, i.e., age (≤40 years vs. >40 years; *p* for the interaction = 0.4595), gender (*p* for the interaction = 0.3303), and BMI (25–28 vs. ≥28 kg/m^2^; *p* for the interaction = 0.5591), significantly modified the association between the serum NPY levels and MUO.

### 3.3. Analysis of the Nonlinear Relationship between NPY and MUO

After we analyzed the association between the MUO phenotype and NPY level using multivariable linear regression and found the results of quartile equidistant variables were not entirely linearly distributed, we considered whether a nonlinear relationship might exist between NPY serum levels and MUO.

**Figure 1:**
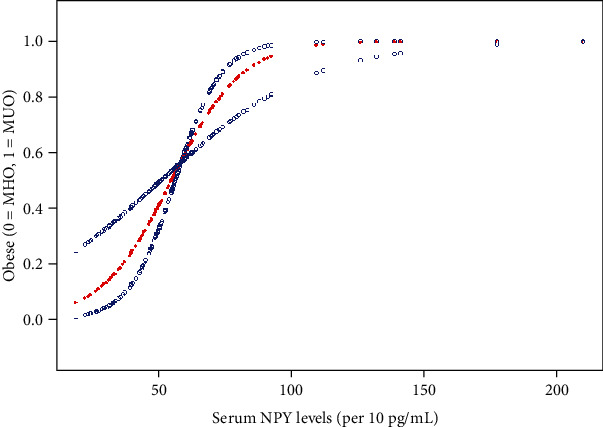
The curved lines illustrate the relationship between the serum NPY levels and MUO. A threshold and nonlinear association between serum NPY levels (per 10 pg/mL increment) and MUO was found (*p* = 0.001). The area between the two dotted lines represents the 95% CI. The blue bands represent the 95% CI for the smoothed curve fit, and the red line represents the smoothed curve fit between the variables. The model was adjusted for sex, age, BMI, WC, WHR, TC, LDL-C, and HbA1c.

Therefore, we performed smooth curve fitting after adjusted for sex, age, BMI, WC, WHR, TC, LDL-C, and HbA1c. A generalized additive model was used, and a threshold and nonlinear association was found between serum NPY levels and MUO (*p* = 0.0001, Figure 1). Threshold saturation effects were further analyzed, and the data showed that the inflection point was 471.5 pg/mL (Table 5). Specifically, no significant relationship was found between MUO and NPY when the serum NPY level was before the turning point (OR: 1, 95% CI: 0.90–1.11, *p* = 0.9920). When serum NPY level was above the inflection point, each 10 pg/mL increment in the NPY level was significantly correlated with an 18% increased OR of the MUO phenotype (OR: 1.18, 95% CI: 1.07–1.29, *p* = 0.0007).

Finally, the specific correlation between NPY and clinic-metabolic indicators in MUO population was analyzed. The results showed that NPY serum level was significantly positively correlated with TG level, but not significantly correlated with other indexes (Table S1).

## 4. Discussion

Our study showed the serum NPY level was remarkably higher in participants with MUO than in those with MHO. NPY was identified as a significant risk factor for MUO in this study's sample of Chinese participants who were obese. We also found a threshold effect based on the serum NPY level, and the risk of MUO associated with NPY increased significantly when the NPY serum level exceeded the threshold. To our knowledge, this may be the first epidemiologic study to provide evidence for a nonlinear relationship between NPY and MUO.

Obesity can increase the risk of some chronic diseases [1, 6, 31], and increasing number of researches have found that NPY contributes to the occurrence and development of obesity and its associated metabolic diseases through its role in the central stimulation of appetite and the regulation of peripheral fat metabolism [13, 17, 20, 21]. Several studies also have reported a link between NPY and obesity. Nyström et al. [32] found that plasma NPY levels were positively correlated with TC and LDL-C serum levels in women and that NPY may be a possible gender-specific cardiovascular risk marker. Karvonen et al. [22] reported a link between cholesterol metabolism and NPY. Ilhan et al. [24] reported that T2DM patients had increased serum NPY levels, which positively correlated with insulin levels. Several studies also showed [23, 33] that the NPY rs16147 genotype could influence changes in abdominal adiposity in overweight or obese participants with dietary interventions. However, the obese populations involved in these studies were not stratified further. Based on the combination of metabolic abnormalities in obese individuals, obesity can be divided into MHO phenotype and MUO phenotype [5, 9].

There were significant differences between the two phenotypes in anthropometric and biochemical parameters, as shown in our study. Among these obese participants, MUO were more likely to be old compared to MHO, coincident with the research by Vasim et al. [34]. What's more, the risk of cardiovascular events among the MUO population is significantly higher than in the MHO population [1]. As for this orexigenic peptide, it is not clear whether these two obese phenotypes in these studies had the same NPY concentrations as no data on NPY serum levels were reported for individuals with MHO and MUO. In the current study, we found increased serum NPY levels in MUO participants, which was partly consistent with the previous reports of elevated NPY levels in T2DM patients [24]. As obesity-related metabolic abnormalities include not only glucose metabolic disorders but also dyslipidemia and hypertension, our results further support the positive association between NPY and metabolic disturbances reported in the existing research literature [24]. As far as our findings are concerned, although the specific mechanism remains unclear, there may be several plausible explanations for the observation elevated in serum NPY levels in the MUO participants.

First, high levels of NPY could lead to hyperphagia, which has been reported to cause a variety of metabolic diseases, such as hyperlipidemia and T2DM. Blood NPY might also come from the NPY stored in the sympathetic ganglion of the digestive tract, as ingestion stimulates the release of NPY into the blood [35]. Chronic hyperlipidemia, in turn, leads to the proliferation of NPY-containing nerve fibers in the digestive tract, leading to an increase in NPY synthesis [36]. Therefore, serum NPY synthesis can be elevated in obese participants with hyperlipidemia. Second, NPY can be secreted by adipocytes located in visceral adipose tissue and increased by insulin stimulation [12, 37]. Adipose-derived NPY can impair the insulin sensitivity of adipocytes [38] and increase the circulating levels of NPY in obese patients [39]. Short-term injections of NPY into the lateral ventricle of mice have been reported to increase their levels of insulin; specifically, the NPY inhibited endogenous glucose and induced insulin resistance [40], while the effect of diabetes-induced appetite enhancement was attenuated afterwards in NPY-targeted knockout mice [41].

The stratified analysis showed a distinct positive association between NPY levels and being male in the MUO sample. Given that Zahorska-Markiewicz et al. [42] reported lower NPY plasma concentrations among women with extreme obesity (BMI = 38.3 ± 4.4) compared with healthy subjects, there might be gender differences in terms of the relationship between NPY and obesity. It is worth noting that a possible reason for the weak association with being female in our study may be the relatively small proportion of the female participants. The *p* value of the later interaction analysis was not significant, indicating that the results of the different stratifications were consistent, and the results of the present study were reliable.

We conducted further multivariate regression to analyze the specific association between NPY and MUO. Our data showed that serum NPY levels were strongly associated with MUO, whether in the form of a continuous variable or a classified variable, after adjusting for the related confounders. Surprisingly, the present study showed a possible nonlinear relationship between serum NPY levels and MUO phenotype for the first time, by smooth curve fitting analysis. We found that NPY did not necessarily have a significant positive association with MUO. Only when its serum level exceeded the inflection point (471.5 pg/mL) was the increased NPY level associated with a higher risk of MUO. In other words, a high level of NPY in MHO individuals may predict the risk of subsequent metabolic disorders, such as glucose metabolism dysfunction, hyperlipidemia, and hypertension. Our previous studies found that different NPY levels had a distinct role in the proliferation and differentiation of adipocytes, and only at high concentrations can NPY promote lipid droplet synthesis and adipogenesis [13]. Therefore, from the clinical point of view, the data of our study confirmed the important value of NPY concentration segmentation.

We also analyzed the correlation between NPY level and metabolic indexes in MUO obese people. Our results showed that TG was the only index that positively correlated with NPY, which were partly consistent with the previous studies showing that NPY signal can regulate TG release, and injection of NPY into the central nervous system can increase both serum TG level and liver TG level [20, 43].The regulatory effect of NPY on TG release may be related to the activities of enzymes involved in phospholipid synthesis, and this process is controlled by the sympathetic nerve of the liver [43]. Therefore, we speculated that the expression of phospholipid synthesis-related enzymes in MUO population may be significantly higher than that in MHO population, which needs to be further confirmed in future study.

The highlight of the present study was the stratification of obesity based on its related metabolic disorders in the analysis of NPY levels in obese participants. Compared with the goal of achieving a normal weight by losing a lot of weight, moderate weight loss might be a short-term and more achievable goal for obese individuals, as it is sufficient for the transition from the MUO phenotype to the MHO phenotype [1]. Obesity and its related metabolic abnormalities are closely related to dietary intake and the resulting energy imbalance, which is largely regulated by NPY [10]. Therefore, the analysis of the distribution of serum NPY expression in MHO and MUO participants can directly reflect the correlation between this orexigenic peptide and MUO; our data additionally underscore the potential involvement of NPY in the pathogenesis of metabolic disorders related to obesity. Furthermore, multivariate analysis and smooth curve fitting helped us find a threshold effect in the relationship between NPY and MUO phenotype. As far as we know, research on the relationship between MUO and the distinct levels of NPY has not been conducted until now. NPY serum levels in obese subjects with MHO phenotype were relatively lower than those with MUO phenotype, but it had exceeded the inflection point (471.5 pg/mL) that was first observed in this study. Since the NPY level at this level was positively associated with the MUO risk, our results provide new evidence on the view that people with MHO are still at risk for developing MUO [1, 6, 7]. A higher NPY level in participants with the MHO phenotype might serve as marker indicating a risk of occurrence of MUO in the future.

There were several limitations of the study that merit mention. First, the defining criteria for MHO are not uniform worldwide [9]; hence, the results of our present study may differ from other studies depending on variations in the defining criteria used. Second, we did not collect data on body fat distribution for some reason, which has been found to be closely associated with obesity. Third, the participants in this study were mainly from Hunan province, so the conclusion can be mainly applicable to population in southern China, while may not be fully applicable to population in other parts of China such as northern China and western China. Additionally, we cannot draw a causal conclusion that increased NPY serum levels promote MUO development in obese Chinese adults in present study, thus, it may be meaningful and necessary to conduct a prospective research in the future. Finally, it was worth mentioning that NPY exerts biological effects via its receptors and the most well-known being NPY-Y1, NPY-Y2, and NPY-Y5 receptors which are involved in obesity [44, 45]. Since these receptors were highly distributed in the hypothalamus and peripheral tissues [44, 45], we will detect their serum levels in the future study.

## 5. Conclusions

In conclusion, the present study suggests that there are significant differences in serum NPY levels in obese participants with different metabolic conditions, and there is a significant association between serum NPY level and MUO. We found a nonlinear relationship between the MUO phenotype and serum NPY levels in the sample of obese Chinese adults, and higher serum NPY levels were positively associated with metabolic abnormalities in these obese individuals.

## Figures and Tables

**Table 1:** Baseline characteristics of the study participants (*N* = 164).

Characteristic	All (*n* = 164)	Obesity		*p* ^∗^
		MHO (*n* = 71)	MUO (*n* = 93)	
Age (years)	44.53 (14.04)	39.94 (13.09)	48.03 (13.78)	<0.001
Sex				0.488
Male	120 (73.0%)	50 (70.4%)	70 (75.3%)	
Female	44 (27.0%)	21 (29.6%)	23 (24.7%)	
BMI (kg/m^2^)	28.11 (2.51)	27.78(2.38)	28.50 (2.46)	0.061
WC (cm)	92.15 (6.80)	89.23 (4.50)	94.39 (7.41)	<0.001
WHR	0.94 (0.06)	0.93 (0.06)	0.96 (0.06)	0.003
SBP (mmHg)	133.24 (18.66)	121.68 (12.17)	142.06 (17.96)	<0.001
DBP (mmHg)	81.66 (11.90)	75.08 (8.76)	86.69 (11.56)	<0.001
TG (mmol/L)	1.67 (1.12-2.78)	1.19 (0.90-1.37)	2.35 (1.73-3.33)	<0.001
TC (mmol/L)	4.83 (1.13)	4.49 (0.70)	5.09 (1.32)	<0.001
HDL-C (mmol/L)	1.18 (0.30)	1.32 (0.24)	1.08 (0.31)	<0.001
LDL-C (mmol/L)	2.88 (0.93)	2.63 (0.65)	3.07 (1.05)	<0.001
FBS (mmol/L)	5.45 (5.05-7.14)	5.08 (4.78-5.29)	6.46 (5.54-9.18)	<0.001
NEFA (mmol/L)	0.49 (0.25)	0.45 (0.15)	0.51 (0.29)	0.174
HbA1C (%)	5.60 (5.30-6.67)	5.30 (5.10-5.50)	6.40 (5.60-7.90)	<0.001
NPY(pg/mL)	585.95 (257.93)	478.89 (145.53)	667.69(292.90)	<0.001

Data were reported as the mean (SD) or median (IQR: Q1-Q3). ^∗^The *t*-test or Mann-Whitney *U* test or *χ*^2^ test was used for comparisons between MHO group and MUO group.

MHO: metabolically healthy obese; MUO: metabolically unhealthy obese; WC: waist circumference; WHR: waist to hip measurement ratio; BMI: body mass index; SBP: systolic blood pressure; DBP: diastolic blood pressure; FBS: fasting blood sugar; TG: triglycerides; TC: total cholesterol; HDL-C: high-density lipoprotein-cholesterol; LDL-C: low-density lipoprotein-cholesterol; NEFA: nonesterified fatty acids.

**Table 2:** Univariate analysis of variables of the participants with MUO.

Variable	OR (95% CI)	*p*
Age (years)	1.05 (1.02–1.07)	0.0004
Sex		
Male	1.0	
Female	0.78 (0.39–1.57)	0.4881
BMI (kg/m^2^)	1.18 (1.02–1.35)	0.0234
WC (cm)	1.16 (1.09-1.24)	<0.0001
WHR (per 1SD)	1.64 (1.16-2.30)	0.0048
SBP (mmHg)	1.10 (1.06–1.13)	<0.0001
DBP (mmHg)	1.12 (1.08–1.16)	<0.0001
TG (mmol/L)	5.30 (2.93–9.61)	<0.0001
TC (mmol/L)	1.69 (1.24–2.32)	0.0011
HDL-C (mmol/L)	0.04 (0.01–0.15)	<0.0001
LDL-C (mmol/L)	1.77 (1.22–2.58)	0.0029
FBS (mmol/L)	11.81 (4.54–30.69)	<0.0001
NEFA (mmol/L)	4.18 (0.48–36.23)	0.1940
HbA1c (per 1%)	51.80 (11.37–235.97)	<0.0001
NPY(per 10 pg/mL)	1.06 (1.03–1.08)	<0.0001

ORs are given per year increase in age, for male vs. female, per 1 SD in WHR, per 1% in HbA1c, per 10 pg/mL in NPY, and per 1 unit in the other parameters (i.e., BMI, WC, SBP, DBP, TG, TC, HDL, LDL, FBS, and NEFA) with 95% CIs.

**Table 3:** Multivariate analysis of the association of serum NPY levels with MUO.

Exposure	Model 1		Model 2		Model 3		Model 4	
	OR (95% CI)	*p*	OR (95% CI)	*p*	OR (95% CI)	*p*	OR (95% CI)	*p*
NPY levels								
Per 10 pg/mL increment	1.06 (1.03-1.08)	<0.0001	1.06 (1.03-1.08)	<0.0001	1.08 (1.04-1.12)	0.0003	1.07 (1.03-1.12)	0.0010
Quartile 1	1.0		1.0		1.0		1.0	
Quartile 2	2.05 (0.84-5.04)	0.1171	2.35 (0.90-6.14)	0.0822	3.11 (0.70-13.73)	0.1346	2.44 (0.51-11.58)	0.2628
Quartile 3	2.75 (1.12-6.78)	0.0278	2.91 (1.11-7.59)	0.0292	5.41 (1.15-25.39)	0.0324	3.93 (0.77-20.07)	0.0994
Quartile 4	19.92 (5.86-67.71)	<0.0001	22.55 (6.29-80.87)	<0.0001	38.99 (6.18-246.01)	<0.0001	29.85 (4.38-203.62)	0.0005
*p* trend	<0.0001		<0.0001		<0.0001		<0.0001	

Model 1 was unadjusted; model 2 was adjusted for sex and age; model 3 was adjusted for model 2 plus BMI, WC, and WHR; and model 4 was adjusted for model 3 plus TC, LDL-C, and HbA1c. Quartile 1: 189.32–460.89 pg/mL, quartile 2: 460.89–559.45 pg/mL, quartile 3: 559.45–659.34 pg/mL, and quartile 4: 659.34–2097.09 pg/mL.

**Table 4:** Effect size of NPY on MUO in subgroups.

Characteristic	Effect size (95% CI)	*p*	*p* for interaction
Sex			0.3303
Male	1.09 (1.03–1.16)	0.0039	
Female	1.05 (0.98–1.12)	0.1906	
Age (years)			0.4595
≤45	1.09 (1.03–1.15)	0.0041	
>45	1.05 (0.98–1.13)	0.1603	
BMI (kg/m^2^)			0.5591
25–28	1.06 (1.01–1.12)	0.0166	
≥28	1.09 (1.02–1.16)	0.0078	

The effect size of association was quantified by OR and 95% CI. Adjusted for sex, age, BMI, WC, WHR, TC, LDL-C, and HbA1c except the subgroup variable.

**Table 5:** Analysis of the threshold effect of NPY on MUO.

Inflection point of NPY (per 10 pg/mL increment)	Effect size (OR)	95% CI	*p*
<471.5	1.00	(0.90–1.11)	0.9920
≥471.5	1.18	(1.07–1.29)	0.0007

The associated effect size was quantified by OR and 95% CI. Adjusted for sex, age, BMI, WC, WHR, TC, LDL-C, and HbA1c.

## Data Availability

The raw data supporting the conclusions of this study will be made available by the authors.

## Abbreviations

NPY: Neuropeptide Y
MHO: Metabolically healthy obesity
MUO: Metabolically unhealthy obesity
T2DM: Type 2 diabetes mellitus
BMI: Body mass index
WC: Waist circumference
WHR: Waist to hip measurement ratio
SBP: Systolic blood pressure
DBP: Diastolic blood pressure
FBS: Fasting blood sugar
TG: Triglyceride
TC: Total cholesterol
HDL-C: High-density lipoprotein cholesterol
LDL-C: Low-density lipoprotein cholesterol
NEFA: Nonesterified fatty acid
IQR: Interquartile range
OR: Odds ratio
CI: Confidence interval.

## Supplementary Materials

Supplementary Materials Table S1: correlations of clinical parameters and metabolism indexes with NPY levels in obese subjects with MUO phenotype.
