# Supplementary material for: Higher Serum Neuropeptide Y Levels Are Associated with Metabolically Unhealthy Obesity in Obese Chinese Adults: A Cross-Sectional Study
Source: Mediators Inflamm. 2020 Aug 4;2020:7903140. doi: 10.1155/2020/7903140 (PMC7424399; doi:10.1155/2020/7903140)
Supplement: Supplementary Materials — Table S1: correlations of clinical parameters and metabolism indexes with NPY levels in obese subjects with MUO phenotype. [file 7903140.f1.docx]

Table S1 Correlations of clinical parameters and metabolism indexes with NPY levels in obese subjects with MUO phenotype.

| Variable |  | *rho* | *P value* |
| --- | --- | --- | --- |
| Age |  | -0.124 | 0.237 |
| BMI |  | 0.199 | 0.056 |
| WC |  | 0.006 | 0.955 |
| WHR |  | -0.060 | 0.566 |
| SBP |  | -0.025 | 0.808 |
| DBP |  | 0.100 | 0.339 |
| TG |  | 0.360 | 0.000 |
| TC |  | 0.184 | 0.077 |
| HDL-C |  | 0.157 | 0.133 |
| LDL-C |  | 0.028 | 0.793 |
| NEFA |  | 0.173 | 0.155 |
| FBS |  | 0.005 | 0.959 |
| HbA1c |  | -0.025 | 0.814 |
